# Evaluation of the therapeutic effect of high-flow nasal cannula oxygen therapy on patients with aspiration pneumonia accompanied by respiratory failure in the post-stroke sequelae stage

**DOI:** 10.1186/s12890-020-01359-5

**Published:** 2021-01-07

**Authors:** Dong Xing, Yu-Hong Chen, Lan -Tao Wang, Bin Yu, Zhi -Bin Ran, Li Chen

**Affiliations:** 1 Department of Emergency, The Fourth Hospital of Hebei Medical University, Shijiazhuang, 050011 China; 2 Intensive Care Unit, The Fourth Hospital of Hebei Medical University, Shijiazhuang, 050011 China; 3 Department of Neurology, Shijiazhuang Great Wall Hospital, Shijiazhuang, 050000 China; 4 Department of General Medicine, The Fourth Hospital of Hebei Medical University, No. 12 of Jiankang Road, Chang’an District, Shijiazhuang, 050011 China

**Keywords:** Stroke, Aspiration pneumonia, Respiratory failure, Venturi mask, High-flow nasal cannulae oxygen

## Abstract

**Background:**

The aim of the present study was to evaluate the therapeutic effect of high-flow nasal cannula (HFNC) oxygen therapy on patients with aspiration pneumonia accompanied by respiratory failure in the post-stroke sequelae stage, with the goal of providing more effective oxygen therapy and improving patient prognosis.

**Methods:**

Retrospective analysis was conducted on 103 elderly patients with post-stroke aspiration pneumonia and moderate respiratory failure (oxygenation index: 100–200 mmHg) that had been admitted. The patients were divided into two groups according to the mode of oxygen therapy that was used: the Venturi mask group and the HFNC treatment group. The two groups were analyzed and compared in terms of the changes in the blood gas indices measured at different points in time (4, 8, 12, 24, 48, and 72 h), the proportion of patients that required transition to invasive auxiliary ventilation, and the 28-day mortality rate.

**Results:**

A total of 103 patients were retrospectively analyzed; 16 cases were excluded, and 87 patients were included in the final patient group (42 in the HFNC group and 45 in the Venturi group). There was a statistically significant difference in the oxygenation indices of the HFNC group and the Venturi group (F = 546.811, *P* < 0.05). There was a statistically significant interaction between the monitored oxygenation indices and the mode of oxygen therapy (F = 70.961, *P* < 0.05), and there was a statistically significant difference in the oxygenation indices for the two modes of oxygen therapy (F = 256.977, *P* < 0.05). HFNC therapy contributed to the improvement of the oxygenation indices at a rate of 75.1%. The Venturi and HFNC groups also differed significantly in terms of the proportion of patients that required transition to invasive auxiliary ventilation within 72 h (*P* < 0.05). The HFNC group’s risk for invasive ventilation was 0.406 times that of the Venturi group (*P* < 0.05). There was no statistical difference in the 28-day mortality rate of the two groups (*P* > 0.05).

**Conclusion:**

HFNC could significantly improve the oxygenation state of patients with post-stroke aspiration pneumonia and respiratory failure, and it may reduce the incidence of invasive ventilation.

## Background

Approximately 15 million people worldwide experience a new or recurrent stroke each year, and up to two thirds of these individuals suffer permanent disability or death [1, 2]. Dysphagia is very common in patients that have experienced a stroke, with an incidence rate of 30–81%. Although the majority of patients with dysphagia may spontaneously recover after a period of rehabilitation treatment, other patients still have the sequelae of dysphagia 6 months after the stroke. There is evidence that complications are the primary cause of morbidity and mortality after an acute stroke [3, 4]. The injury to the patients’ neurological function affects the oropharynx and esophagus, which results in saliva, drinking water, and food flowing back to the respiratory tract and causing pulmonary inflammatory disease. Respiratory failure caused by aspiration pneumonia is a major cause of death after a stroke and accounts for approximately 35% of patient deaths. For most patients with long-term dysphagia, the incidence and mortality rates of aspiration pneumonia related to dysphagia will increase in the absence of high quality care, resulting in poor patient prognosis [5]. Although aspiration pneumonia only accounts for 5–15% of community-acquired pneumonia cases, it develops rapidly and has a high mortality rate in comparison with other types of pneumonia. Previous studies have found that patients with respiratory failure caused by aspiration pneumonia have a 5-year mortality rate of 18.7%, which is higher than the mortality rate of other types of pneumonia [6, 7]. Previous studies have also observed that aspiration is the most important and direct cause of respiratory failure in patients that have experienced a stroke [8]. The main treatment of aspiration pneumonia follows antibiotic therapy with oxygen therapy to quickly correct the patients’ systemic oxygenation state and avoid the subsequent occurrence of multiple organ dysfunction syndrome (MODS)—the simultaneous occurrence of two or more organ dysfunctions or failure.

In daily clinical practice, aspiration pneumonia in patients with stroke is often considered to be caused by pulmonary infection as a result of aspiration. Aspiration pneumonia after a stroke can have many serious complications, including airway obstruction, exogenous lipoid pneumonia, diffused bronchiolitis, and pulmonary abscess. In addition, patients that have experienced a stroke have lower cough and expectoration reflexes and a limited self-expectoration ability. Although effective antibiotic and oxygen therapies have been used clinically, the problems of anoxia and high mortality have not been solved [9, 10]. Patients with acute respiratory distress syndrome (ARDS), which is characterized by severe hypoxia and diffused inflammatory infiltration of the pulmonary tissue, require longer hospital stays, incur higher treatment costs, and experience a poor quality of life and a high mortality rate [11, 12]. Therefore, it is necessary to find a mode of oxygen therapy that can quickly correct the respiratory failure caused by aspiration pneumonia and, as a result, improve the prognosis of patients after a stroke.

As modern oxygen therapy technology continues to progress, high-flow nasal cannula (HFNC) oxygen therapy has been widely used in patients that experience acute respiratory failure after post-surgery extubation. In those patients, HFNC has been shown to significantly reduce the incidence of re-intubation, lower the cost of treatment, shorten the length of stay, and improve patient prognosis [13]. However, few studies have considered the use of HFNC in patients with respiratory failure after a stroke. The purpose of the present study was to explore how a more effective mode of oxygen therapy can improve the prognosis of patients with aspiration pneumonia and respiratory failure. The study retrospectively analyzed the changes in oxygenation status and differences in outcome in patients that underwent different oxygen therapies to treat aspiration pneumonia and respiratory failure after a stroke.

## Methods

### General materials

The study enrolled 103 elderly patients with post-stroke aspiration pneumonia and moderate respiratory failure (Oxygenation index (PO2/FiO2): 100–200 mmHg) that had been admitted to the Emergency Department of the Fourth Hospital of Hebei Medical University between November 2018 and November 2019. Patients met the following diagnostic criteria of aspiration pneumonia [14]: patients had aspiration, X-ray or computerized tomography imaging showed that the new pulmonary infiltrative lesions were in accordance with the inflammatory changes, and patients had any two of the following symptoms: (1) an increase in leukocyte or neutrophil counts as indicated by routine blood tests; (2) fever, with a body temperature > 38.3 °C or an increase of ≥ 1 °C; (3) cough and expectoration of an increased amount of phlegm with a change in nature; and (4) signs of moist rales in the lungs or lung consolidation.

The inclusion criteria were as follows: patients over 65 years old; patients with more than one stroke attack (including hemorrhagic and ischemic stroke) at least 24 weeks prior; patients with moderate hypoxemia (oxygenation index: 100–200 mmHg) at the time of the first blood gas analysis after admission to the emergency room; patients with the results of blood gas analysis at set points after admission (0, 4, 8, 12, 24, 48, and 72 h); and patients admitted for at least 72 h of observation, or admitted for hospitalization. The exclusion criteria were as follows: patients with aspiration pneumonia not caused by stroke; patients with serious disturbance of consciousness (coma); patients with endotracheal intubation upon admission or emergency endotracheal intubation within 8 h of admission; patients whose family did not allow regular blood gas analysis; and patients with incomplete data. The present study was reviewed and approved by the ethics committee of the hospital.

### Analytic methods

The patients were divided into the Venturi mask group and the HFNC group. Monitoring by electrocardiogram and finger pulse oxygen monitor was performed in both groups immediately after admission. In the HFNC group, the flow velocity was set at 50 L/min, the humidification temperature was set at 37 °C, and the oxygen concentration was adjusted at 21–100%. In the venturi group, the oxygen flow was adjusted according to the patient's condition, and the oxygen concentration was adjusted at 21–50%. The oxygen concentrations in the two groups were adjusted to keep the level of oxygen saturation above 90%. When the level of oxygen saturation could not maintained above 90%, nasotracheal intubation was performed on the patient.

General clinical indicators were collected for both groups, including age, gender, type of stroke, state of consciousness, and accompanied organ failure. Blood gas analysis was used to evaluate the oxygenation status and was carried out immediately after admission (0 h) and again at 4, 8, 12, 24, 48, and 72 h after admission. Data were also collected for both groups concerning the 28-day mortality rate and the proportions of patients that required transition to invasive auxiliary ventilation at different times.

### Statistical methods

The SPSS 21.0 software package (IBM Corp., USA) was used for the statistical analysis of the data. Normally distributed measurement data were expressed as mean ± standard deviation (SD), and the comparisons were examined by Student-t test and Mann–Whitney test (non-parametric distribution). The categorical data were expressed as n (%), and the differences between the two groups were examined by chi-square analysis or Fisher's exact test. *P* < 0.05 was considered statistically significant.

## Results

In the present study, 103 patients were retrospectively analyzed; 16 cases were excluded, leaving 87 patients for final inclusion in the analysis. The HFNC group comprised 42 patients, and the Venturi group comprised 45 patients. The general clinical data of all patients were statistically analyzed using the χ2 test. Eighteen patients (42.9%) in the HFNC group and 31 patients (68.9%) in the Venturi group had a stroke duration > 48 weeks, the difference in the two groups was statistically significant (*P* < 0.05). In the Venturi group, 29 patients (64.4%) were complicated with MODS, and the involved organs included 45 cases (100%) of lung, 8 cases (17.8%) of kidney, 7 cases (15.6%) of circulation, 5 cases (11.1%) of blood and 9 cases (20.0%) of gastrointestinal tract. In HFNC group, 15 patients (35.7%) were complicated with MODS, and the involved organs included 45 cases (100%) of lung, 6 cases (14.3%) of kidney, 4 cases (9.5%) of circulation, 1 case (2.4%) of blood, and 4 cases (9.5%) of gastrointestinal tract. There was a significant difference in the proportion of MODS between the two groups (*P* < 0.05). Both groups of patients were sent to the rescue room because of respiratory failure caused by pulmonary infection. There were 19 patients (45.2%) with chronic obstructive pulmonary disease (COPD) in HFNC group and 9 patients (20%) with COPD in Venturi group, the difference in the two groups was statistically significant (*P* < 0.05). There was no significant difference in the proportion of patients with chronic left heart failure or diabetes mellitus between the two groups (*P* > 0.05) (Table 1). The results of the arterial blood gases analysis showed that there were significant differences in PaCO2 between the two groups measured at 4 h, 8 h and 12 h after admission (*P* < 0.05), but there was no significant difference in PaCO2 measured at 0 h, 24 h, 48 h and 72 h after admission (*P* > 0.05) (Table 1).

**Table 1:** Statistic results of the general clinical characteristics between the two groups

Observation indexes	Venturi group N = 45	HFNC group N = 42	*χ*2 OR *t* value	*P* value
Female, n (%)*	23 (51.1)	22 (52.4)	0.014	0.906
Age ≥ 75 years old, n (%)*	21 (46.7)	25 (59.5)	1.441	0.230
The proportion of those with multiple organ failure n (%)^Δ^	29 (64.4)	15 (35.7)	7.174	0.007
Involved organ				
Lung	45 (100)	42 (100)		
Kidney	8 (17.8)	6 (14.3)		
Circulation	7 (15.6)	4 (9.5)		
Blood	5 (11.1)	1 (2.4)		
Gastrointestinal	9 (20.0)	4 (9.5)		
Co-morbidities				
Chronic left heart failure (NYHA III or IV), n (%)*	10 (22.2)	13 (40.0)	0.851	0.356
Diabetes mellitus, n (%)*	22 (48.9)	18 (42.9)	0.318	0.573
Chronic obstructive pulmonary disease (COPD), n (%)^Δ^	9 (20)	19 (45.2)	6.340	0.012
State of somnolence, n (%)*	23 (51.1)	18 (42.9)	0.594	0.441
Duration of stroke^Δ^			5.984	0.014
≥ 48 weeks, n (%)	31 (68.9)	18 (42.9)		
< 48 weeks, n (%)	14 (31.1)	24 (57.1)		
(Rehabilitative therapies 48 weeks after stroke)*			0.900	0.343
Yes, n (%)	19 (42.2)	22 (52.4)		
No, n (%)	26 (57.8)	20 (47.6)		
PaCO_2_(mmHg) mean (SD)				
0H	47.4 (5.4)	46.8 (5.7)	− 0.308	0.761
After 4 h*	41.6 (3.6)	37.6 (2.9)	− 3.361	0.002
After 8 h^Δ^	39.1 (2.7)	35.5 (2.0)	− 4.080	0.000
After 12 h^Δ^	37.0 (1.7)	35.5 (1.4)	− 2.598	0.014
After 24 h*	35.3 (1.9)	34.8 (2.5)	− 0.547	0.588
After 48 h*	35.5 (1.6)	35.4 (1.6)	− 0.214	0.832
After 72 h*	36.2 (1.9)	35.9 (1.4)	− 0.521	0.606
Ischemic stroke, n (%)*	16 (35.6%)	17 (40.5%)	0.223	0.636

*No statistical difference between the two different therapeutic groups (*P* > 0.05)

^Δ^With a statistic difference between the two different therapeutic groups (*P* < 0.05)

**Fig. 1:**
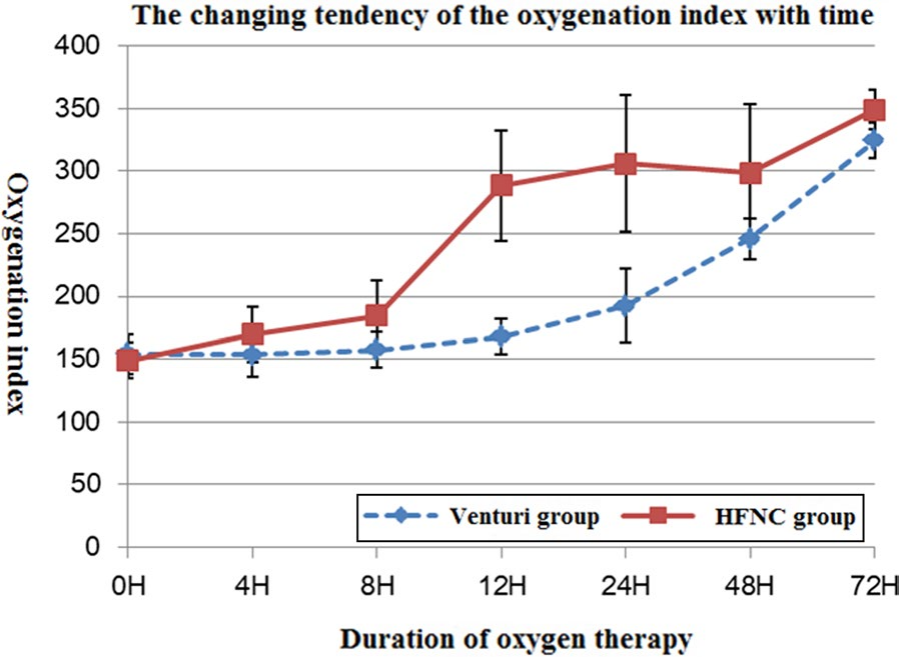
The changing tendency of the oxygenation index with time

A two-factor repeated measures ANOVA was used to analyze the changes in the oxygenation indices of the two groups as measured at different points in time. The ANOVA results demonstrated that there was a significant difference in the oxygenation indices of the HFNC and Venturi groups as measured at different points in time (F = 546.811, *P* < 0.05). There was also a statistically significant interaction between the monitored oxygenation indices and the mode of oxygen therapy (F = 70.961, *P* < 0.05). HFNC therapy contributed to the improvement of the oxygenation indices at a rate of 75.1%. There were statistically significant differences in the oxygenation indices of the two groups under different modes of oxygen therapy, as measured over time (F = 256.977, *P* < 0.05) (Fig. 1).

The two groups were also compared in terms of the 28-day mortality rate and the proportion of patients that required transition to invasive auxiliary ventilation. The results showed that there was no statistically significant difference between the two groups in the incidence of invasive ventilation after receiving endotracheal intubation at 12H and 72H (*P* > 0.05). The incidence of invasive ventilation after receiving endotracheal intubation at 24H and 48H in Venturi group was significantly higher than that in the HFNC group (*P* < 0.05). Within 72 h, the total incidence of invasive ventilation with endotracheal intubation was higher in the Venturi group than that in the HFNC group (*P* < 0.05), and the risk of invasive ventilation was relatively lower in the HFNC group. However, there was no statistically significant difference in the 28-day mortality rate of the two groups (*P* > 0.05) (Table 2).

**Table 2:** The proportion of patients with transition to the invasive auxiliary ventilation (%) and the 28-day mortality between the two groups

	Venturi group	HFNC group	*χ*2 value	*P* value	*OR*	95% *CI*
Invasive auxiliary ventilation^a^						
12H	3 (6.7%)	6 (14.3%)	1.360	0.244	0.429	0.100–1.837
24H	12 (40.0%)	3 (9.1%)	7.560	0.006	6.222	1.539–25.153
48H	8 (36.4%)	2 (6.5%)	7.521	0.006	8.286	1.551–44.264
72H	3 (15.8%)	4 (14.9%)	0.008	0.928	1.078	0.212–5.488
Total	26 (63.4%)	15 (41.3%)	4.244	0.039	0.406	0.171–0.964
28-day mortality^b^	6 (11.9%)	5 (13.3%)	0.000	1.000	0.878	0.247–3.125

^a^With a statistic difference in the proportion of patients with transition to the invasive auxiliary ventilation between the two groups (*P* < 0.05)

^b^No difference in the 28-day mortality between the two groups (*P* > 0.05)

## Discussion

Aspiration pneumonia combined with respiratory failure is a common cause of death in patients that have experienced a stroke. The primary treatment of this condition is to rapidly correct the hypoxia state, improve the oxygen supply to the tissue, and use antibiotics to prevent MODS from hypoxia [15]. HFNC is a new type of oxygen therapy that not only can provide relatively stable oxygen concentrations and highly efficient airway humidification but also can regulate the flow velocity to provide positive end-expiratory pressure in the respiratory tract, similar to that which would be provided by a ventilator. HFNC is also better tolerated and has a better treatment effect than traditional invasive modes of oxygen therapy [16, 17]. Studies from many countries have shown that, in addition to significantly improving oxygenation status and tolerance, HFNC can reduce the incidence of re-intubation in patients with chronic obstructive pulmonary disease [18, 19]. HFNC has also played an important role in the treatment of viral pneumonia, including MERS-CoV pneumonia, H1N1 pneumonia, and novel coronavirus pneumonia. It has become part of a relatively standardized treatment protocol and has achieved a good therapeutic effect [20]. The traditional mode of oxygen therapy often has a poor effect for patients that experience complications from aspiration pneumonia after a stroke: Injury to their central nervous system leads to limited respiratory function and a reduced ability to discharge phlegm autonomously, and these patients will inevitably require invasive auxiliary ventilation, increasing their medical burden. In addition, the prognosis of patients that also experience ventilator-associated pneumonia is very poor.

To improve the oxygenation status of patients and avoid tracheal intubation, it is necessary to identify a non-invasive mode of oxygen therapy that is more effective than traditional modes of oxygen therapy. Through the retrospective analysis of 87 patients with post-stroke aspiration pneumonia and respiratory failure, it was found that HFNC significantly improved the patients’ oxygenation status and was especially effective for the early and rapid correction of hypoxia. As shown by the contour map (Fig. 1), the oxygenation indices of the HFNC group began to improve approximately 4 h after therapy, and the improvement in oxygenation indices after 8–24 h was significantly greater than in the traditional Venturi mask treatment group. Repeated measurement statistics demonstrated that HFNC therapy contributed to the improvement of oxygenation indices at a rate of 75.1%.

Meanwhile, we found that there were different degrees of CO_2_ retention in the two groups of patients, in addition to the pulmonary infection during admission, some patients with COPD also aggravated the probability of CO_2_ retention. Interestingly, although the proportion of patients in the HFNC group combined with COPD was significantly higher than that in the Venturi group, with the implementation of the two oxygen therapies, the CO_2_ retention rate of the HFNC group compared with Venturi group was significantly improved at about 4–12 h. We mainly considered two factors: firstly, HFNC was a new type of nasal humidification oxygen supply method, which could output constant oxygen concentration in the range of 21–100% under the condition of constant temperature of 37 °C and relative humidity of 100%, so as to ensure the normal function of airway mucosa cilia, promote sputum dilution, and effectively ensure the discharge of airway secretions. The maximum oxygen flow output of HFNC could reach 60 L/min, which could effectively increase the alveolar ventilation volume of patients, and reduce the ventilation volume of the physiological invalid alveolar cavity, at the same time, it could reduce the respiratory power consumption in the inspiratory phase, quickly and effectively alleviate the symptoms of dyspnea. The oxygen inhalation device could also generate a certain positive airway pressure in the expiratory phase, similar to the positive end expiratory pressure (PEEP: 2–5 cm H_2_O), which could promote the expansion of atelectasis, increase the residual capacity of the lung and counteract endogenous PEEP, while correcting hypoxemia, CO_2_ retention was also reduced. However, the Venturi mask was made according to the Venturi principle. When oxygen entered the mask through a narrow hole, a negative pressure was generated around the jet airflow, and a certain amount of air was carried into the mask from the open edge. The size of the mask edge seam changed the ratio of air to oxygen. Its main advantage was that it could humidify oxygen and provide stable oxygen concentration (up to 50%), and high flow rate gas can effectively avoid repeated inhalation of CO_2_. Therefore, compared with the two oxygen therapy methods, HFNC may be more effective in improving lung function, promoting oxygenation and reducing CO_2_ retention in the same oxygen concentration [16, 17]. Secondly, the traditional view indicated that the improvement of neurological function mainly occurs in the 12–24 weeks after stroke and tends to be stable. However, recent studies have shown that if timely and effective rehabilitation treatment was given after stroke, about a quarter of ischemic stroke patients could continuously improve their neurological function within 12–48 weeks after stroke, thus improving the 5-year clinical outcomes of stroke patients [21]. On the contrary, if the patients could not get effective rehabilitation treatment, or the recovery of neurological function was not ideal after treatment, as the disease progresses, the incidence of aspiration pneumonia caused by stroke induced systemic immune suppression and improper feeding was significantly increased. And with the extension of stroke duration, the patient's condition would develop into severe oxygenation disorder due to pulmonary infection, then develop into multiple organ failure, and the mortality rate of 1–5 years was significantly increased.[22, 23]. In this study, there was a significant difference in duration of stroke (≥ 48 weeks) between the two groups, which might have an impact on the oxygenation state and proportion of patients with multiple organ failure. In addition, we compared the incidence of invasive ventilation after receiving endotracheal intubation at different time cutoff points (12 h, 24 h, 48 h, 72 h) and the total incidence of invasive ventilation with endotracheal intubation in 72 h between the two groups. The results showed that before 12 h, there was no significant difference in the incidence rate of invasive ventilation between the two groups. In 24–48 h, the invasive ventilation rate in the Venturi group was higher than that in the HFNC group; in 48–72 h, the incidence of invasive ventilation was not statistical different. This result is consistent with the improvement trend of oxygenation in patients (Fig. 1).

In addition, it was also found that, within 72 h after treatment, a significantly lower proportion of patients from the HFNC group required invasive ventilation than in the Venturi group, where the incidence of invasive ventilation in the HFNC group was 0.406 times than of the Venturi group. This suggests that HFNC could significantly reduce the incidence of transition to invasive auxiliary ventilation. The possible reason was that with HFNC treatment, the atelectasis of lung tissues were effectively reversed, and the oxygenation status was improved, however, some severe non-functional lung tissues did not have the basis for functional improvement under the existing time and treatment conditions. However, as far as the overall observation window (72 h) was concerned, HFNC could significantly reduce the incidence of invasive ventilation compared with Venturi treatment in such patients.

Although the incidence of multiple organ failure was significantly different between the two groups [Venturi group: 29 (64.4%) vs HFNC: 15 (35.7%)], the 28-day mortality rate of the two groups showed no obvious statistical difference. The reasons may be as follows: for the patients with multiple organ failure, we not only give them positive respiratory function support, but also give multiple organ comprehensive treatment measures including continuous blood purification and nutritional support, which may play a positive role in reducing the mortality of patients.

The analysis of the general data for the patients in the two groups also revealed differences in terms of organ failure. A higher proportion of patients experienced MODS in the Venturi group than in the HFNC group. A study by Ping et al. [24] found that the improvement of 90-day neurological function in patients that had experienced an acute stroke could be attributed to the improvement of oxygenation of organs, including brain tissue, during the early post-stage. However, the study did not specifically analyze the time at which the patients’ hypoxic state was significantly corrected, and all of the enrolled patients were in the acute stage of stroke (within 24 h of onset) and had not experienced organ failure. By contrast, the patients enrolled in the present study were all in the post-stroke sequelae stage, and some were experiencing MODS. As a result, this study found significantly higher rates of mortality and invasive auxiliary ventilation than were reported in the study mentioned above.

Since the 90’s, noninvasive ventilation (NIV) has been widely used in COPD exacerbation but with controversial results aspiration pneumonia combined with respiratory failure [25]. NIV can create a much higher gas flow rate and reduce inspiratory effort through positive pressure. Compared to NIV, HFNC is simpler to use and apply, at the same time, HFNC is more comfortable and delivers high fraction of inspired oxygen, and HFNC generates a low level of positive pressure [26]. HFNC can also provide irrigation of dead space in the upper respiratory. Therefore, HFNC is more preferred than NIV in the treatment of patients with aspiration pneumonia combined with respiratory failure [27, 28].

The present study had some limitations. First, the results may have been impacted by the sample size. Second, the results may have been biased because the study was not stratified according to the specific organs that failed, the number of organs that failed, or the duration of stroke sequelae. The results must be verified by a stratified randomized controlled study.

## Conclusion

Compared with the Venturi mask, HFNC oxygen therapy can improve the oxygenation status of patients with aspiration pneumonia and respiratory failure in the post-stroke sequelae stage and reduce the incidence of invasive auxiliary ventilation. However, HFNC therapy was found to have no effect on the 28-day mortality rate.

## Data Availability

We declared that materials described in the manuscript, including all relevant raw data, will be freely available to any scientist wishing to use them for non-commercial purposes, without breaching participant confidentiality. If anyone wish to obtain the study data, please contact the corresponding author: Li Chen.

## Abbreviations

HFNC: High-flow nasal cannula
ARDS: Acute respiratory distress syndrome
